# Experimental datasets on the extraction of functional ingredients from seaweeds for controlling bacterial infection

**DOI:** 10.1016/j.dib.2025.111569

**Published:** 2025-04-21

**Authors:** Nehal M. El-Deeb, Tamer A.E. Ahmed, Amal D. Premarathna, Vitalijs Rjabovs, Rando Tuvikene, Riadh Hammami, Martine Boulianne, Maxwell T. Hincke

**Affiliations:** a Department of Cellular and Molecular Medicine, Faculty of Medicine, University of Ottawa, Ontario K1H 8M5, Canada; b Pharmaceutical Bioproducts Research Department, City of Scientific Research and Technological Applications (SRTA-City), New Borg El-Arab City, Alexandria, Egypt; c School of Natural Sciences and Health, Tallinn University, Narva mnt 29, 10120 Tallinn, Estonia; d National Institute of Chemical Physics and Biophysics, Akadeemia tee 23, 12618 Tallinn, Estonia; e Institute of Chemistry and Chemical Technology, Riga Technical University, Paula Valdena Iela 3/7, LV-1048 Riga, Latvia; f School of Nutrition Sciences, Faculty of Health Sciences, University of Ottawa, Ontario K1H 8M5, Canada; g Department of clinicat sciences, Faculté de médecine vétérinaire, Université de Montréal, Québec G5L 3A1, Canada; h Department of Innovation in Medical Education, Faculty of Medicine, University of Ottawa, Ontario K1H 8M5, Canada

**Keywords:** *Ascophyllum nodosum*, Ulva lactuca, Antibacterial, Phenolics and flavonoids

## Abstract

Seaweeds are gaining significant attention for their bioactive compounds, which hold great potential for use in food, cosmetics, and pharmaceuticals [1]. To avoid the use of toxic substances in the extraction process, there is a need for innovative and eco-friendly methods to exploit the highly potent raw seaweed biomass. Described herein are the datasets of how the particle size reduction of seaweeds positively enhanced the efficacy of green extraction in boosting the extraction yields of seaweed bioactive compounds.

Different green extraction approaches were used to accumulate different seaweed particle sizes that were collected via grinding and sieving [2]. The total yields of carbohydrates, glucuronic acids, proteins, phenolics and flavonoids were quantified to evaluate the efficacy of the extraction strategies. The efficacy and safety usages of the extracts were assessed using different pathogenic bacterial strains and human cell lines, respectively.

Specifications Table

**Table:** 

Subject	Health and Medical sciences
Specific subject area	Infectious disease and nutrition.
Type of data	*Tables and figures*.
Data collection	Particle size collection: Dried seaweed samples (*Ulva lactuca - Chlorophyta, and Ascophyllum nodosum - Phaeophyceae*) were ground and sieved, and then subjected to various types of heated aqueous extraction protocols. Extraction optimization was evaluated by biochemical and phytochemical analyses. Antibacterial activity (planktonic and biofilm) and safety profiling assays were performed. Seaweed extract constituents were identified by amino acid and lipid profiling.
Data source location	The extraction procedures and the biochemical and phytochemicals evaluations were conducted at the Department of Cellular and Molecular Medicine, Faculty of Medicine, University of Ottawa, Ontario, K1H 8M5, Canada. GPS coordinates: 45.7419° N, 73.9893° W. The biological evaluation and the safety assessment experiments were completed at the School of Nutrition Sciences, Faculty of Health Sciences, University of Ottawa, Ontario, K1H 8M5, Canada. GPS coordinates: 45.7419° N, 73.9893° W. The Amino acid and lipid profiling was done at the School of Natural Sciences and Health, Tallinn University, Narva mnt 29, 10120 Tallinn, Estonia. GPS coordinates: 59.4408° N, 24.7489° E
Data accessibility	*The data are available here and in data Mendeley data repository at this link*https://data.mendeley.com/datasets/m755r38rhs/1
Related research article	*data Mendeley data repository at this link*https://data.mendeley.com/datasets/m755r38rhs/1.

## 1 Value of the Data

• Superior extraction efficiencies were obtained from various seaweed [*U. lactuca* and *A. nodosum* (G-H)] particle sizes produced by grinding and sieving.
• The biochemical and phytochemical analysis enables the selection of optimal heating conditions based on the carbohydrate, Glucuronic acid, protein, phenolic compounds and flavonoids yields of *U. lactuca* and *A. nodosum*.
• The biological activity assays confirmed the efficacy and safety profile of the extracts produced by the optimized protocols, providing researchers with a logical strategy to produce valuable bioactive compounds.
• The amino acid profiling and fatty acids analysis of the extracts allowed the comparison of amino acid and fatty acid contents between the two optimized extracts
• Accessibility and Contribution to the Community: This dataset is made publicly available (https://data.mendeley.com/drafts/m755r38rhs), encouraging collaboration among researchers in the field seaweed extraction provides open access to high-quality data, promoting transparency and knowledge sharing.

## 2 Background

The data presented herein demonstrates that the reduction of seaweed particle size significantly improved the extraction efficacy. This analysis identified the most efficient extraction protocol (after optimizing each protocol in the original research article) based on the extraction yields of total carbohydrates, protein, gluconic acid, phenolics and flavonoids, and evaluated the biological activities and safety profile of the optimized seaweed extracts.

## 3 Data Description

**Fig. 1:**
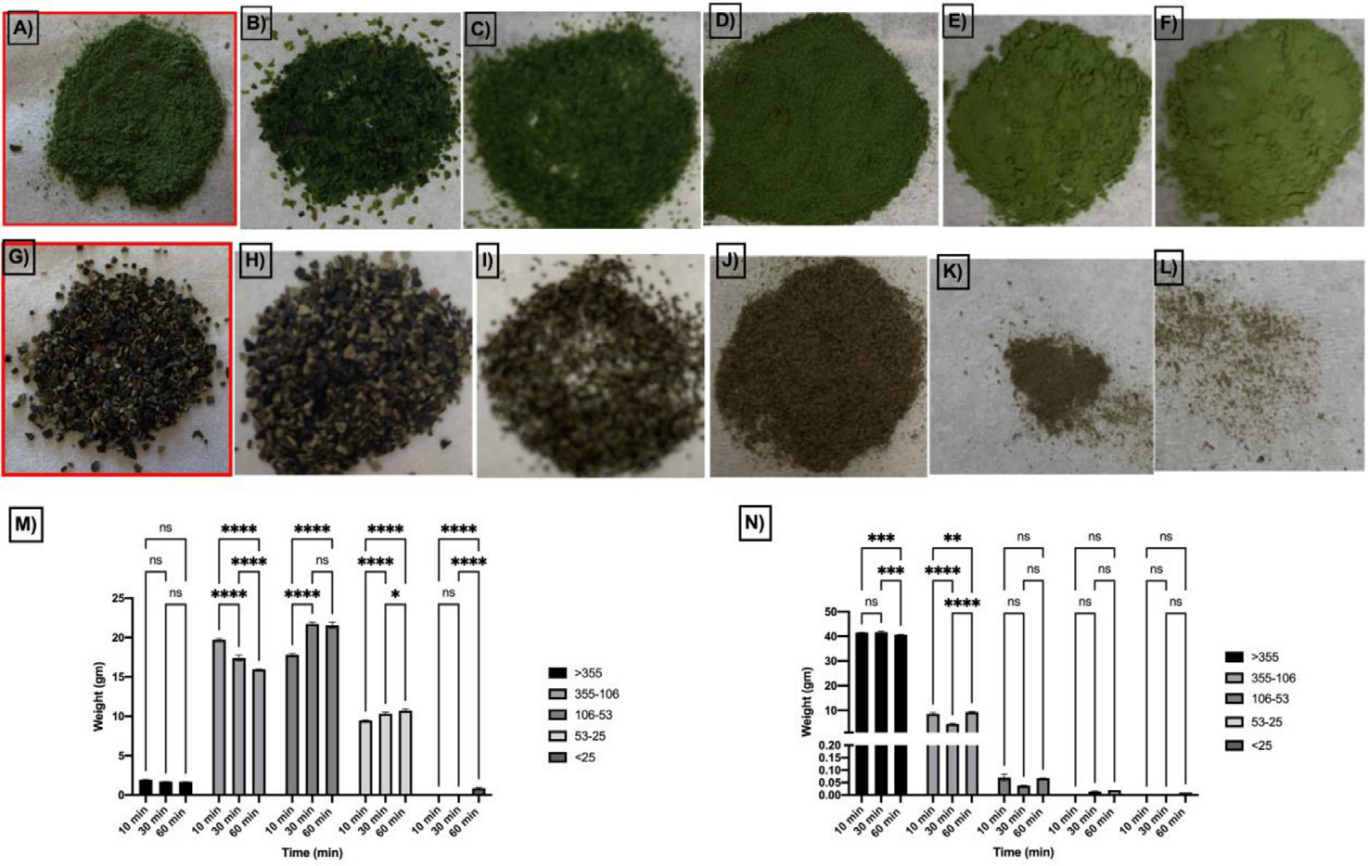
Distribution of algal biomass across fractions.

**Fig. 2:**
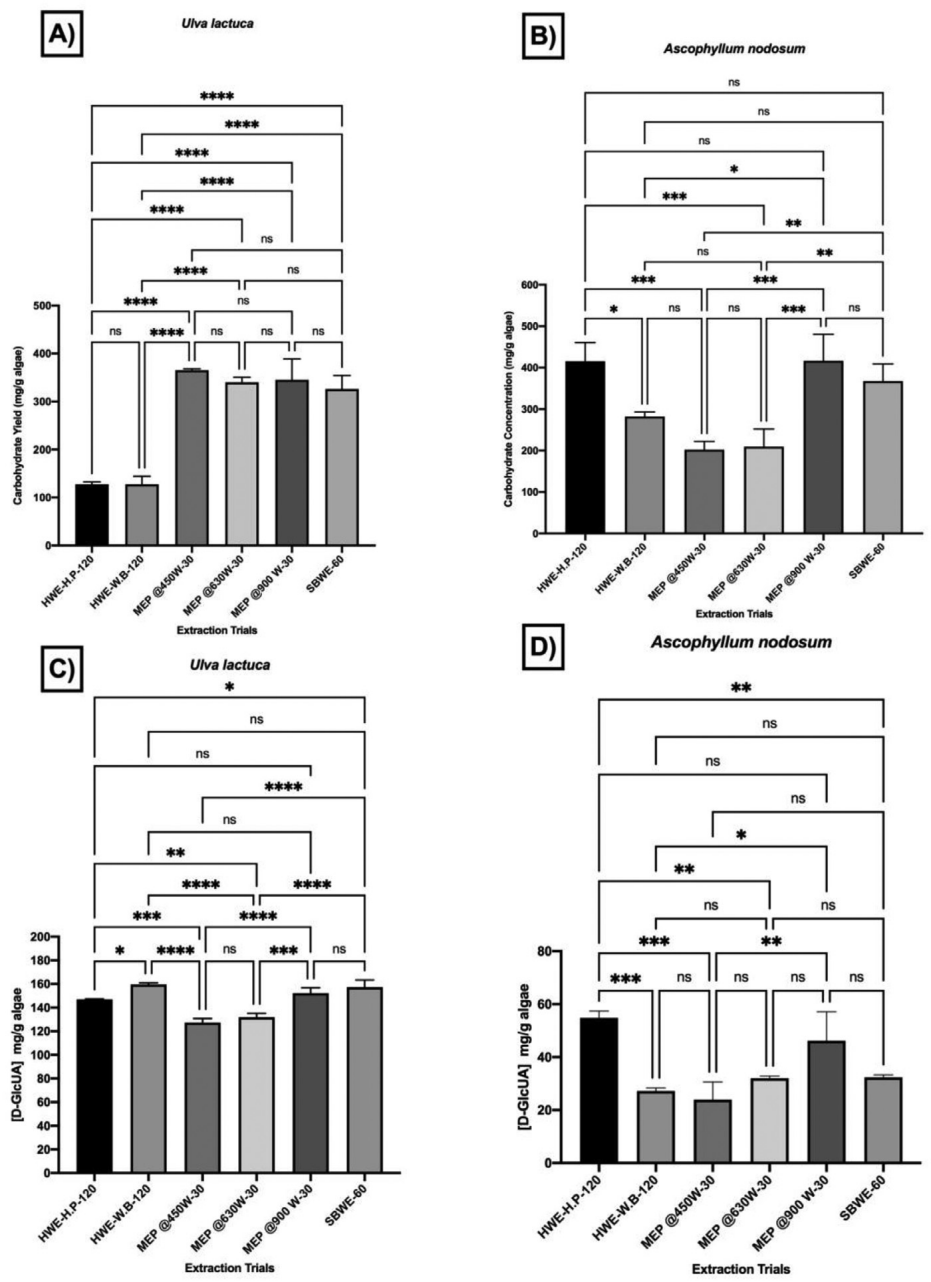
Comparison of the optimized heating conditions on the carbohydrate and Glucuronic acid yields for *Ulva lactuca* and *Ascophyllum nodosum*. The total carbohydrate results of 25–53 µm particle size were replotted for each extraction protocol, enabled the selection of the optimum extraction protocol for *U. lactuca* (MEP@450 for 30 min) (A) and *A. nodosum* (MEP@900 for 30 min) (B). In addition, the total glucuronic acid results for 25-53 µm particle size were replotted for each extraction protocol, enabled the selection of the optimum extraction protocol for *U. lactuca* (MEP@900 for 30 min) (C) and *A. nodosum* (D). All the assays were performed in triplicate (n=3). Two-way ANOVA and the multiple comparisons were done using GraphPad Prism 8 where ns: non-significant at adjusted *P* value 0.999, **: Significant at adjusted *P* value 0.0022 and *** Significant at adjusted *P* value <0.0001.

**Fig. 3:**
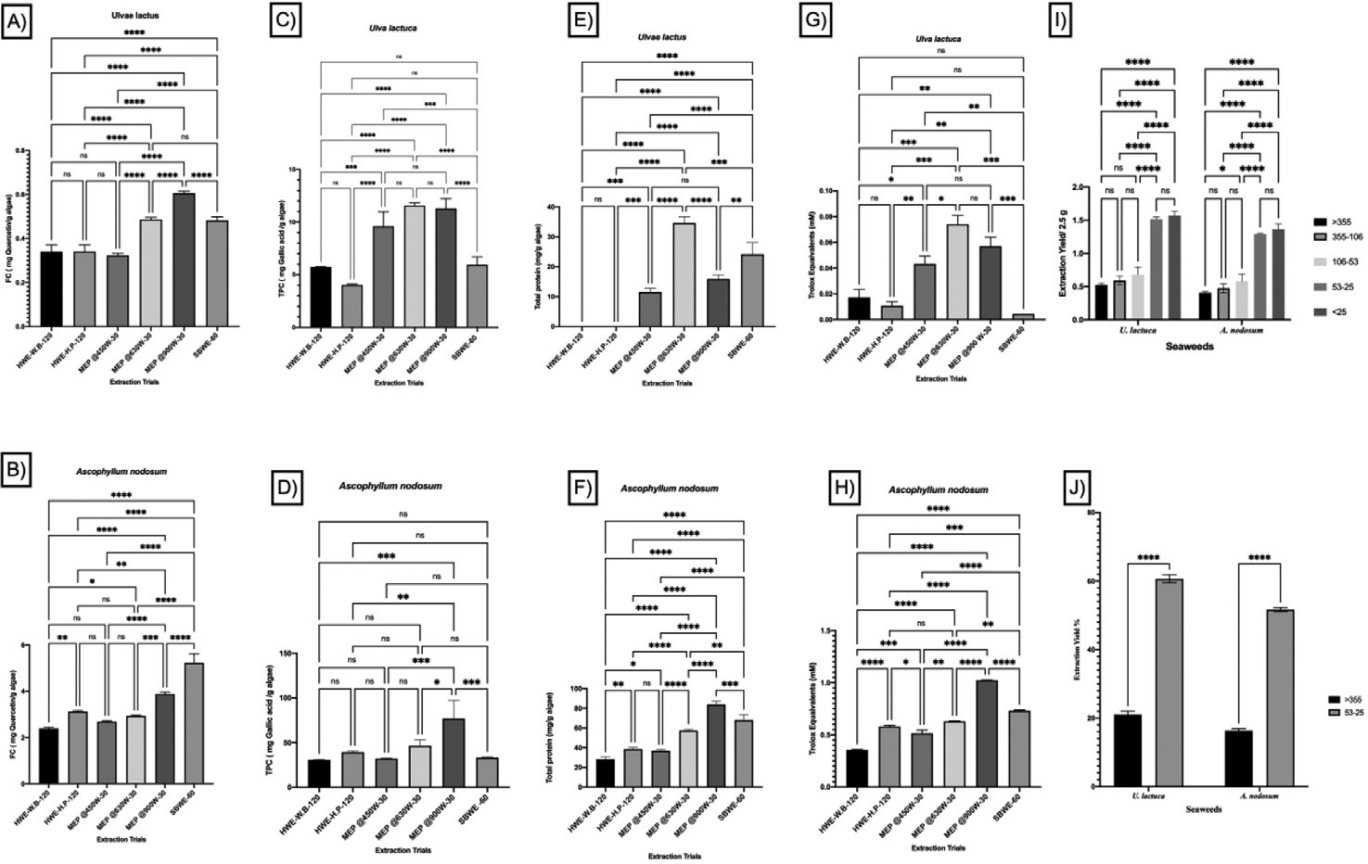
Comparison of the effect of optimized heating conditions on flavonoid, total phenolics, protein yields, antioxidant activities and the extraction yield of *U. lactuca* and *A. nodosum*. The total flavonoid (A, B), total phenolics (C, D), and protein yields (E, F) for 25-53 µm particle size were replotted for each extraction protocol in order to select the optimum extraction protocol. The optimum extraction conditions for total flavonoids including MEP@6300 for 30 min for *U. lactuca* (A), and MEP@900 for 30 min for *A. nodosum* (B). In addition, the optimum extraction protocol for total phenolics included MEP@900 for 30 min for *U. lactuca* (C) and *A. nodosum* (D). The optimum extraction protocols for protein were identified as (MEP@6300 for 30 min) for *U. lactuca* (E) and (MEP@900 for 30 min) for *A. nodosum* (F). All the assays were performed in triplicate (n=3). Two-way ANOVA and the multiple comparisons were done using GraphPad Prism 8 where, ns: non-significant at adjusted *P* value 0.997, *: Significant at adjusted *P* value 0.0173 **: Significant at adjusted *P* value 0.0035 and *** at adjusted *P* value 0. 0004. To clarify how the particle size reduction enhanced the antioxidant powers of the seaweed extracts using the above-mentioned protocols, we plotted the antioxidant activity results of 25-53 µm particle sizes with >355 µm results. Then, the antioxidant activity result of 25-53 µm fraction for each extraction protocol was collected and plotted together to statistically analyze them to select the optimum extraction protocol for *U. lactuca* (G), and *A. nodosum* (H). Furthermore, a comparison of the amount of bioactive extract from different particle sizes for *U. lactuca*, and A. nodosum was done (I, J). The weights (g) obtained for extracts from 2.5vg of seaweed biomass of different particle sizes (I). Comparison of extraction efficiency between coarse (>355 µm) and fine (25-53 µm) particle sizes, for the optimized protocols that were applied to each seaweed (J). All the assays were performed in triplicate (n=3). Two-way ANOVA and the multiple comparisons were done using GraphPad Prism 8 where, ns: non-significant at adjusted *P* value 0.719, *: Significant at adjusted *P* value 0.0446, **: Significant at adjusted *P* value 0.0011 and *** Significant at adjusted *P* value <0.0001.

**Fig. 4:**
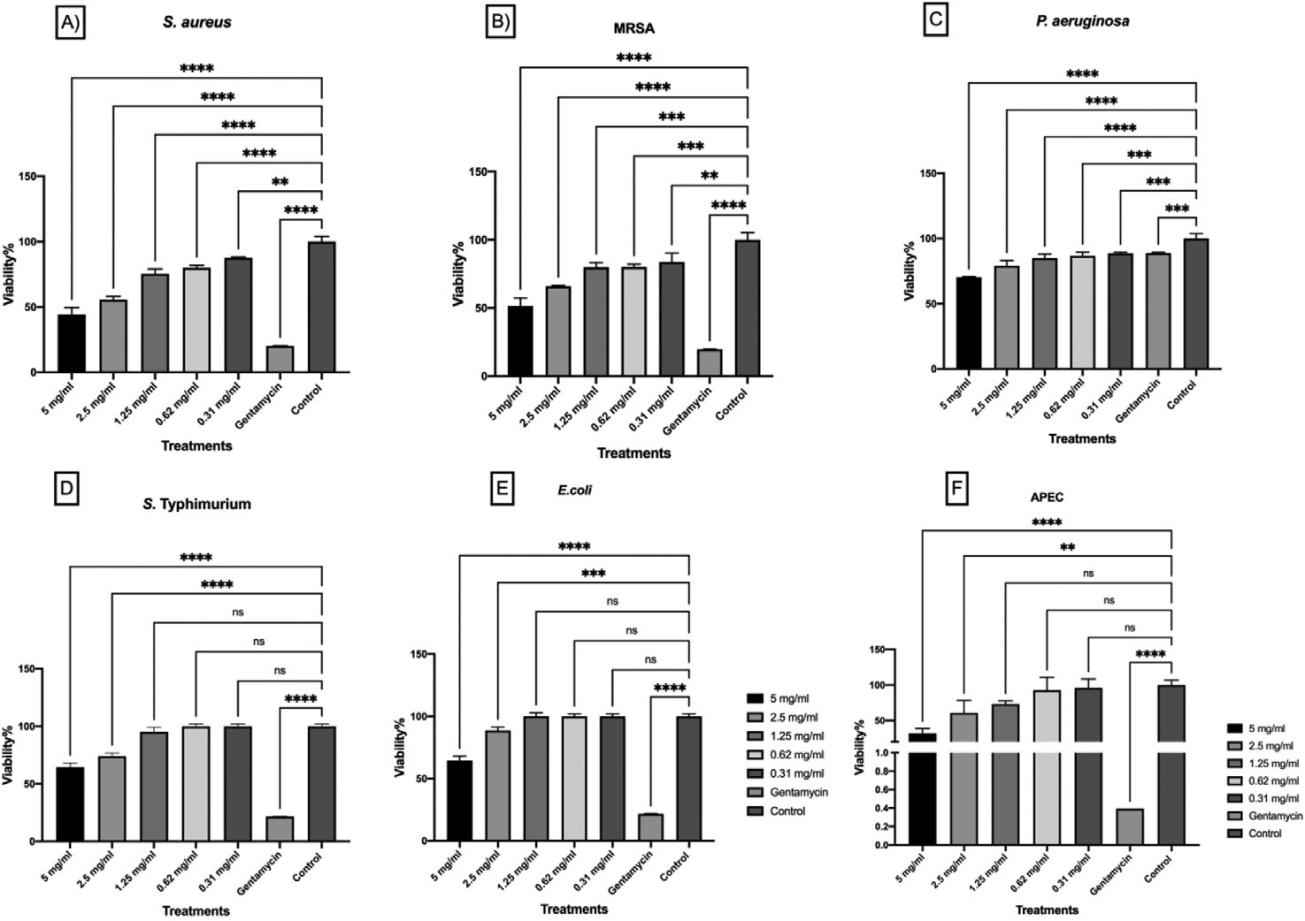
The effects of different seaweed extracts on the viability of human pathogenic bacteria. The antibacterial activities of the optimized extracts (*U. lactuca* (U-ME): 25-53 µm particle size prepared with MEP@6300 for 30 min; and for *A. nodosum* (A-ME): 25-53 µm particle size, prepared with MEP@900 for 30 min) were tested against different bacterial pathogens. A-ME and U-ME extracts were tested against gram-positive bacteria, including *Staphylococcus aureus* (A), and MRSA (B) and gram-negative bacteria including, *Pseudomonas aeruginosa* (C), *Salmonella* Typhimurium (D), *E. coli* (E) and the Avian Pathogenic *Escherichia coli* strain (APEC) (F). The bacterial viability percentages were calculated at the ends of incubation with different treatment concentrations (5–0.15 mg/mL), where the wells with bacterial inoculum without seaweed extracts of antibiotic were considered as a negative control, and wells with LB media and seaweed extracts without bacteria were used as the blank. The bacterial viability % calculated as the following: % Bacterial viability= (A600 Treatment- A600 Blank)/ (A600 Control-A600 Blank) X100. All the assays were performed in triplicate (n=3). Two-way ANOVA and the multiple comparisons were done using GraphPad Prism 8 where, ns: non-significant at adjusted *P* value 0.752, *: Significant at adjusted *P* value 0.0173 **: Significant at adjusted *P* value 0.0033 and **** at adjusted *P* value <0.0001.

**Fig. 5:**
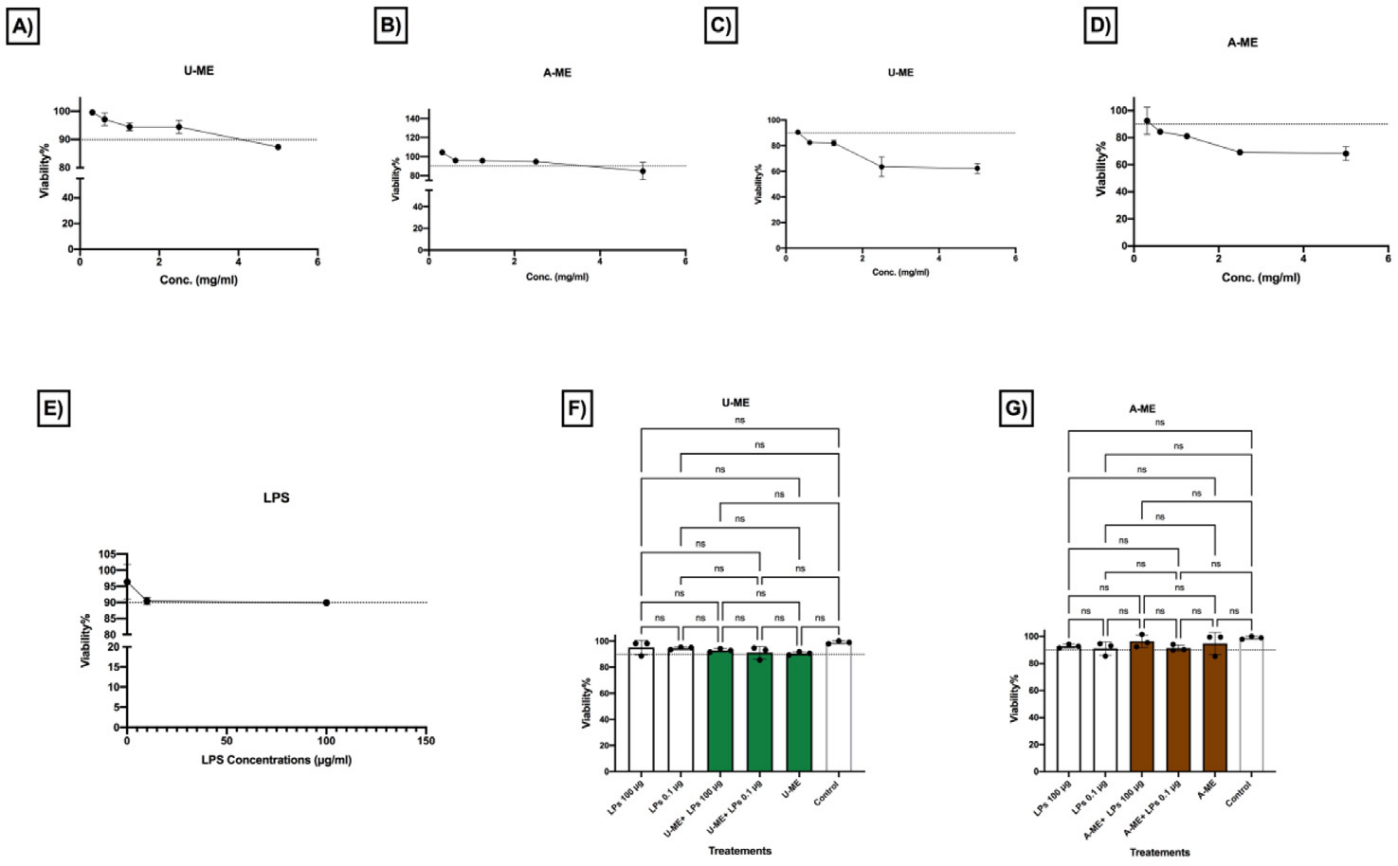
Safety profile of the optimized seaweed extracts evaluated on Caco-2 and macrophage cells.

**Fig. 6:**
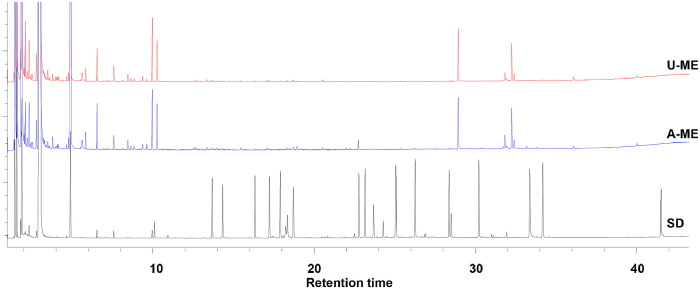
Amino acid profiles of the A-ME, U-ME extracts. SD = amino acid standards.

**Fig. 7:**
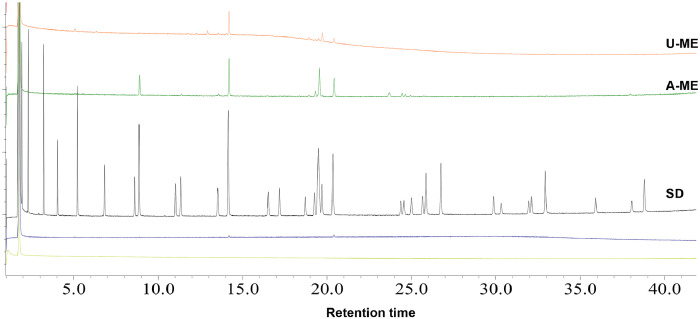
Fatty acid profiles of the A-ME, U-ME extracts. SD = fatty acids standards.

Different seaweed particle sizes were produced by grinding 50 g lots of dried seaweed samples*; U. lactuca* (Fig. 1A) and *A. nodosum* (Fig. 1 G), followed by sieving. Particle sizes ranged from fine to coarse (<25, 25–53, 106–53, 106–355, and >355 µm) from *U. lactuca* (Fig. 1B–F), and *A. nodosum* (Fig. 1H–L), and were generated in different proportions (Fig. 1M, N; respectively). The flowchart in Fig. 2 explains the strategy for selection of the optimized extraction protocol, based on carbohydrate and glucuronic acid yields of *U. lactuca* (Fig. 2A, C) and *A. nodosum* (Fig. 2B, D). Further optimization was based on the recovery of flavonoids, phenolics and protein (Fig. 3A–H). Extraction yields from 25 to 53 µm and >355 µm particle sizes were compared to confirm the effect of particle size reduction in enhancing the efficacy of the optimized extraction protocols (Fig. 3I–J). Data sets in Fig. 4A–F demonstrate the antimicrobial activities of optimized extracts against different gram-positive bacteria (*Staphylococcus aureu*s, and MRSA) and gram-negative bacteria (*Pseudomonas aeruginosa, Salmonella* Typhimurium, *E. coli* and Avian Pathogenic *Escherichia coli* strain, APEC), respectively. The safety profile of optimized seaweed extracts was confirmed on Caco-2 intestinal and RAW macrophage cells (Fig. 5). Amino acid and fatty acid constituents of *A. nodosum* (A-ME) and *U. lactuca* (U-ME) extracts were identified using the GC-MS method [Tables 1, 2; Fig. 6, Fig. 7].

**Table 1:** Retention time of amino acid standards.

Ret. Time (min)	Short Name	Name
13.659	Ala	Alanine
14.31	Gly	Glycine
16.096	β-Ala	Beta-Alanine
16.302	Val	Valine
17.205	Leu	Leucine
17.882	Ile	Isoleucine
18.697	Pro	Proline
21.703	Tau	Taurine
22.749	Met	Methionine
23.13	Ser	Serine
23.666	Thr	Threonine
25.033	Phe	Phenylalanine
26.228	Asp	Aspartic Acid
26.784	Hyp	Hydroxyproline
27.11	Cys	Cysteine
28.329	Glu	Glutamic Acid
28.466	Orn	Ornithine
28.783	Asn	Asparagine
30.167	Lys	Lysine
30.906	Gln	Glutamine
34.131	His	Histidine
34.132	Tyr	Tyrosine
37.716	Trp	Tryptophan
41.455	Cys	Cystine
45.36	Hcy	Homocysteine

**Table 2:** Retention time of Fatty acids standards.

Retention time, min	µg/mL	Short name	Name
-	**400**	**4:0**	**Methyl butyrate**
1.924	**400**	**6:0**	**Methyl hexanoate**
2.303	**400**	**8:0**	**Methyl octanoate**
3.216	**400**	**10:0**	**Methyl decanoate**
4.036	**200**	**11:0**	**Methyl undecanoate**
5.212	**400**	**12:0**	**Methyl dodecanoate**
6.814	**200**	**13:0**	**Methyl tridecanoate**
8.865	**400**	**14:0**	**Methyl myristate**
8.602	**200**	**14:1 ω5**	**Methyl myristoleate**
11.338	**200**	**15:0**	**Methyl pentadecanoate**
11.023	**200**	**15:1 ω5**	**Methyl pentadecenoate (cis-10)**
14.148	**600**	**16:0**	**Methyl palmitate**
13.529	**200**	**16:1 ω7**	**Methyl palmitoleate**
17.183	**200**	**17:0**	**Methyl heptadecanoate**
16.515	**200**	**17:1 ω7**	**Methyl heptadecenoate (cis-10)**
20.347	**400**	**18:0**	**Methyl stearate**
19.695	**200**	**18:1 ω9 trans**	**Methyl elaidate (trans-9)**
19.489	**400**	**18:1 ω9 cis**	**Methyl oleate (cis-9)**
19.489	**200**	**18:2 ω6 trans**	**Methyl linoelaidate**
19.252	**200**	**18:2 ω6 cis**	**Methyl linoleate**
18.709	**200**	**18:3 ω6**	**Methyl γ-linolenate**
19,489	**200**	**18:3 ω3**	**Methyl linolenate (cis-9,12,15)**
26.737	**400**	**20:0**	**Methyl eicosanoate**
25.856	**200**	**20:1 ω9**	**Methyl eicosenoate (cis-11)**
25.661	**200**	**20:2 ω6**	**Methyl eicosadienoate (cis-11,14)**
25	**200**	**20:3 ω6**	**Methyl eicosatrienoate (cis-8,11,14)**
25.856	**200**	**20:3 ω3**	**Methyl eicosatrienoate (cis -11,14,17)**
24.37	**200**	**20:4 ω6**	**Methyl arachidonate (cis -5,8,11,14)**
24.551	**200**	**20:5 ω3**	**Methyl eicosapentaenoate (cis -5,8,11,14,17)**
29.874	**200**	**21:0**	**Methyl heneicosanoate**
32.932	**400**	**22:0**	**Methyl docosanoate**
32.11	**200**	**22:1**	**Methyl erucate**
31.945	**200**	**22:2 ω6**	**Methyl docosadienoate (cis -13,16)**
30.32	**200**	**22:6 ω3**	**Methyl docosahexaenoate (cis -4,7,10,13,16,19)**
35.914	**200**	**23:0**	**Methyl tricosanoate**
38.798	**400**	**24:0**	**Methyl lignocerate**
38.035	**200**	**24:1**	**Methyl nervonate**

The different seaweed particle size fractions (<25, 25-53, 106-53, 106-355, and >355 µm) were collected from *U. lactuca* (Fig. 1A, the ground initial powder, B-F the collected particle size fractions) and *A. nodosum* (Fig. 1G, the ground initial powder, H-L the collected particle size fractions). The distributions of different particle size fractions in ground seaweed samples (M: *U. lactuca* and N: *A. nodosum*) were determined by the weight of each collected fraction at different time intervals of sieving (10, 30 and 60 min).

The effect of different concentrations (5–0.3 mg/mL) of U-ME (A) and A-ME (B) extracts on the viability of Caco-2 cells (ATCC, HTB-37) line was determined using the Alamar blue assay (Thermo Fisher Scientific). Furthermore, the effect of different concentrations of U-ME (5–0.3 mg/mL) (C), A-ME extracts (5–0.3 mg/mL) (D), LPS (0.1, 10 and 100 µg/mL) (E), mixtures of U-ME (at final concentration 0.3 mg/mL) +LPS (at final concentration of 0.1 and 100 µg/mL) (F), and mixtures of A-ME (at final concentration of 0.3 mg/mL) +LPS (at final concentration of 0.1 and 100 µg/mL) (G) on the viability of RAW 264.7 cell Line (ATCC, TIB-71) was determined using the Alamar blue assay (Thermo Fisher Scientific). Cellular viability (%) was calculated after treatment with different concentrations of seaweed extracts with respect to untreated control cells. All the assays were performed in triplicate (n=3). Two-way ANOVA and the multiple comparisons were done using GraphPad Prism 8 where, ns: non-significant at adjusted *P* value 0.991, *: Significant at adjusted *P* value 0.049, **: Significant at adjusted *P* value 0.0024 and **** Significant at adjusted *P* value <0.0001.

## 4 Experimental Design, Materials and Methods

### 4.1 Seaweed particle size reduction and extraction protocols

Different seaweed particle sizes were produced by grinding (Salton grinder) followed by sieving using a Sieve shaker (Silent sifter II SS-22, Gilson S-22, INC. USA) to obtain particle sizes ranging from fine to coarse fractions (<25, 53-25, 106-53, 355-106 and >355 µm). Different hot extraction protocols (direct heating: (Corning PC-620D, USA), water bath (Precision Scientific Dubnoff Metabolic Shaking Incubator Water Bath), microwave (microwave oven; Camco Inc. General Electric, Canada)and subcritical water extraction (SBWE) by autoclaving) were applied for different durations (10, 20, and 30 min).

### 4.2 Phytochemical analysis

Carbohydrate contents of the freeze-dried collected clear extracts were quantified using the phenol–sulfuric acid method [3]. Total glucuronic acid was quantified using the D-Glucuronic/D-Galacturonic Acid Assay Kit (Megazyme Assay kit (K-URONIC) (Bray, WC, Ireland), according to the instruction manual. Quantification was performed using a microplate reader (EOn, Biotek, Thermo Fisher Scientific, USA).

### 4.3 Total phenolics and flavonoid contents

The Total phenolic content (TPC) was determined by Folin-Ciocalteu (FC) Phenol reagents, using gallic acid standard [4]. Furthermore, the total flavonoid content (TFC) was determined using an Oxiselect Flavonoid assay kit (Cell Biolabs) according to the manufacturer’s instructions.

### 4.4 Antibacterial assay

The antibacterial activity of the extracts obtained from seaweeds using the optimized extraction protocol was tested against different pathogenic bacterial strains (*Staphylococcus aureus*, ATCC 6538 and methicillin-sensitive *Staphylococcus aureus*, ATCC 6538), *Escherichia coli*, O157:H7; *Pseudomonas aeruginosa*, ATCC 15442 and *Salmonella* Typhimurium, ATCC 1535). In addition, Avian Pathogenic *Escherichia coli strain* (APEC, ESC0298)). The final concentrations of seaweed extracts ranged from 5 to 0.15 mg/mL. The plates were then incubated for 24 h, and the EOn microplate spectrophotometer with Gen5 data analysis software was used to monitor the growth of bacteria by measuring the optical density (A) at 600 nm at 1 h intervals for 24 h. Gentamycin at a final concentration of 0.62 µg/mL was used as a positive control. The inhibition percentages of bacterial growth were calculated after 24 h incubation as the following:

$$\begin{array}{cc} & \%\;\text{Inhibition}\;\text{of}\;\text{bacterial}\;\text{growth}\\ & \;=\left(A600\text{nm}\;\text{Treatment}-A600\text{nm}\;\text{Blank}\right)/\left(A600\text{nm}\;\text{Control}-A600\text{nm}\;\text{Blank}\right)\times 100. \end{array}$$

An ultrasonic cleaner water bath (Bransonic 220, USA) was used to prepare the mature bacterial biofilms for evaluation of anti-biofilm activity.

### 4.5 Safety profiling

The safety profiles of seaweed extracts were evaluated on two cell lines (Caco-2, ATCC - HTB-37; RAW 264.7, ATCC - TIB-71) using alamarBlue. The alamarBlue absorbance was measured at 570 nm, with 600 nm as a reference (dual wavelength mode) using an EOn microplate spectrophotometer with Gen5 data analysis software. The test was performed in triplicate.

%Viability=(A570Treatment−A600Blank)/(A600Control−A600Blank)×100

The non-toxic concentrations of each seaweed extract were determined as the concentration at which the cellular viability remained >90 % compared to controls without treatment [5].

The Millicell-ERS resistance system (MilliporeSigma, USA) was used to measure Transepithelial electrical resistance (TEER) of confluent Caco-2 cell cultures.

### 4.6 Analytical methods for amino acid profiling of algal polysaccharides

Amino acid concentrations were determined according to the method described by Adler et al. [6]. Lipid extraction and fatty acid methyl ester quantification were performed using the method outlined by Premarathna et al. [7], with a Shimadzu GCMSQP2010 Ultra gas chromatograph equipped with a mass detector (GC-MS) and a Phenomenex Zebron ZB-5MS capillary column (30 m × 0.25 mm, 0.25 µm film thickness). Amino acid and lipid profiling was done using a Shimadzu GCMSQP2010 Ultra gas chromatograph with a mass detector (GC-MS) using different Amino Acid Standards (analytical standard, AAS18 - Sigma-Aldrich) and fatty acids standards (FAME Mix: Supelco CRM47885 standard mixture).

### 4.7 Analytical methods for lipid profiling of algal polysaccharides

The amino acid content of freeze-dried extracts was analyzed using a Shimadzu GCMSQP2010 Ultra gas chromatography coupled with mass spectrometry (GC–MS) and the method outlined by Premarathna et al. [7]. The Quantification was carried out using analytical standards (Supelco A6407, A6282). The characterization of seaweed extracts was done using iSTM50 FTIR-ATR spectrophotometer (Thermo Fisher Scientific, Waltham, MA, USA), LabSolutions GPC post-run software (Shimadzu, Kyoto, Japan), Shimadzu LC-30AD pump, CBM-20A system controller, RID-10A refractive index detector, and CTO-20AC column oven were used to determine the weight-average molecular weights (Mw) and polydispersity indexes (PDI) of the polysaccharide samples.

## Limitations

*Not applicable.*

## Ethics Statement

*The current work does not involve human subjects, animal experiments, or any data collected from social media platforms.*

## CRediT Author Statement

**Nehal EL-Deeb:** Writing –original draft, Review & editing, Methodology, Investigation, Data curation, Data analysis, Software analysis, Conceptualization. **Tamer A.E. Ahmed:** Editing, Conceptualization, Software, Project administration, Data storage. **Amal D. Premarathna:** Identification of seaweed, Writing. **Vitalijs Rjabovs:** Identification of seaweed, Writing – review & editing. **Rando Tuvikene:** Identification of seaweed, review & editing. **Riadh Hammami:** Reviewing, Supervision. **Maxwell Hincke:** Supervision, Writing – review & editing, Funding acquisition, Conceptualization, Visualization, Validation, Software, Resources, Project administration, Data curation.

## Data Availability

Mendeley DataData sets for " Experimental datasets on the extraction of functional ingredients from seaweeds for controlling bacterial infection" (Original data)
